# Characterization and comparative analysis of the *Escherichia marmotae* M-12 isolate from bank vole (*Myodes glareolus*)

**DOI:** 10.1038/s41598-023-41223-0

**Published:** 2023-08-25

**Authors:** Pavel A. Zhurilov, Pavel A. Andriyanov, Anastasia I. Tutrina, Irina V. Razheva, Elena A. Liskova, Nadezda A. Gladkova, Daria D. Kashina, Ivan V. Yashin, Andrey A. Blokhin

**Affiliations:** Federal Research Center for Virology and Microbiology, Branch in Nizhny Novgorod, 603950 Nizhny Novgorod, Russia

**Keywords:** Bacteriology, Bacterial pathogenesis

## Abstract

The *Escherichia marmotae* is a bacterium of the *Enterobacterales* order, which was first isolated from the Himalayan marmot (*Marmota himalayana*). Recently *E. marmotae* has been shown to cause severe infections in humans. Wild animals were suggested to be a natural reservoir of this bacterium. The present study describes the first case of *E. marmotae* isolation from an apparently healthy wild bank vole (*Myodes glareolus*). Phenotype, as well as genotype-based techniques, were applied to characterize *E. marmotae* M-12 isolate. *E. marmotae* M-12 had the capsule-positive phenotype, high adhesion to human erythrocytes and HEp-2 cells as well as a low invasion into HEp-2 cells. *E. marmotae* M-12 was avirulent in mice. The phylogenomic analyses of *E. marmotae* showed dispersed phylogenetic structure among isolates of different origins. Virulome analysis of M-12 isolate revealed the presence of the following factors: siderophores, heme uptake systems, capsule synthesis, curli and type I fimbriae, flagella proteins, OmpA porin, etc. Comparative virulome analysis among available *E. marmotae* genomes revealed the presence of capsule K1 genes mostly in pathogenic isolates and OmpA porin presence among all strains. We assume that the K1 capsule and OmpA porin play a key role in the virulence of *E. marmotae*. Pathogenesis of the latter might be similar to extraintestinal pathogenic *E. coli*.

## Introduction

*Escherichia marmotae* is a Gram-negative bacterium of the *Enterobacterales* order. This species historically belonged to the “Escherichia cryptic clade V”. The latter were considered environmental members of the *Escherichia* genus with low pathogenicity potential^1^.

*E. marmotae* was first isolated from wild animals in particular from the feces of wild Himalayan marmot (*Marmota himalayana*) and was described in 2015^2^. In 2021 *E. marmotae* was also isolated from the fecal of other marmot species (*Alpine marmot*)^3^. Additionally, GenBank contains information about 3 *E. marmotae* strains isolated from the diaphragm of wild boar. However, farm animals were also shown to be a source of this bacterium. In 2020 *E. marmotae* was isolated from cow rectal feces^4^ and in 2021 *E. marmotae* was isolated from fresh fecal samples from farm healthy hens^5^. Moreover, *E. marmotae* was isolated from companion animals. GenBank contains genome assemblies of *E. marmotae* isolated from the external ear canal of canines and canines' milk. Additionally, the aquatic environment can be a source of *E. marmotae* bacteria. There is information in GenBank of *E. marmotae* isolates originated from downstream freshwater samples of wastewater treatment plants in the UK as well as cases of isolation from the waterline in the USA.

In 2019 Liu et al. performed genomic and molecular characterization of *E. marmotae* from wild rodents, and assumed that wild animals may serve as potential reservoirs of *E. marmotae*. In vitro infection assay was also performed and it was noted that *E. marmotae* may be a potential invasive pathogen for humans and animals^6^.

In 2022 in Norway *E. marmotae* was first reported to cause invasive infections in humans^7^. Audun Sivertsen et al., investigated 4 cases of infection in which *E. marmotae* caused sepsis of unknown origin, postoperative sepsis, and upper urinary tract infection. Notably, all infection cases were considered community-acquired. Phylogenetic analysis of the mentioned isolates showed inherent virulence in multiple lineages of *E. marmotae* including nonhuman strains. It was also shown *E. marmotae* has a large accessory genome indicating its ecological plasticity.

Based on the above, *E. marmotae* was isolated from some wild animals as well as farm animals, and water environments and can cause infections in humans. However, the certain reservoir and pathogenic potential of *E. marmotae* especially key virulence factors remain unclear.

Here we report the case of *E. marmotae* isolation from the lungs of a wild bank vole (*Myodes glareolus*). We performed classic microbiological as well as whole-genome sequencing approaches, including comparative analysis, to characterize *E. marmotae* M-12 isolate. The main focus was on the investigation of the virulence of our isolate using different models as well as comparative virulome analysis of *E. marmotae*.

## Materials and methods

### Ethics statement

Animal experiments were performed according to: (I) The Directive 2010/63/EU of the European Parliament and of the Council of 22 September 2010 on the protection of animals used for scientific purposes; (II) ARRIVE 2.0 guidelines^8,9^. All methods were performed under the relevant guidelines and regulations. None of the rodent species investigated in the present study had protected status. Trapping campaigns were systematically performed with prior explicit agreement from relevant local authorities, and from the owners of the territory where trapping was performed.

All procedures involving animals were approved by the Ethics Committee of the Federal Research Center for Virology and Microbiology (certificate No: IRECAS_01, IRECAS_03).

### Mice capture and autopsy procedures

Wild rodents were captured in June 2021 in the Lyskovsky District of the Nizhny Novgorod region, Russia. Animals were captured in the forests on the right bank of the Volga River (56.070217; 45.332745). We used standard household traps that ensured the instant death of the animals. Once a day, the bait (bread) was moistened with vegetable oil. Traps were checked every morning for night captures, and every afternoon for daily captures. Carcasses of caught animals were delivered to the laboratory at a temperature of 4 ± 2 °C and organs were obtained for bacteriological analysis on the same day.

Internal organs were obtained as described elsewhere^10^. Autopsy and sampling of internal organs were carried out under aseptic conditions, with sterile instruments, followed by rapid flaming after each manipulation with the certain organ. The extraction of the internal organs was performed in the following order: lungs, liver, spleen, mesentery, and intestines. Intestines were extracted lastly to avoid contamination with gut microflora.

### *Escherichia marmotae* M-12 isolation

Previously flamed organ samples were cut into small pieces and were transferred to test tubes with 10 ml of Buffered Peptone Water (BPW) (HiMedia, India) for general enrichment. Cultivation was carried out at 37 °C for 18–24 h. The loopful of BPW was then streaked onto Xylose–Lysine Deoxycholate Agar (XLD Agar) (HiMedia, India) and incubated for 18–24 h at 37 °C. The separate pink and yellow colonies were replated on Nutrient agar (NA) (HiMedia, India) to obtain a pure culture for further research. All isolated cultures were stored in Nutrient broth (NB) (HiMedia, India) with 15% glycerol at − 80 °C.

### Microbiological assays

A routine microscopic examination was performed to establish morphological and tinctorial properties. Primary biochemical and cultural properties were also evaluated: catalase, oxidase, and motility. Detection of bacterial capsule production was performed via the Capsule Stains Kit (HiMedia, India). The determination of catalase activity was carried out using 3% hydrogen peroxide, and the oxidase activity was evaluated through OXItest test strips (Erba Lachema, Czech Republic) following instructions. The determination of motility was carried out on Motility Medium (HiMedia, India) at 37 °C for 24 h. ENTEROtest 24N (Erba Lachema, Czech) was used for M-12 biochemical features evaluation.

### Isolate identification

The 16S rRNA gene sequencing approach was used for isolate identification. DNA was extracted from an 18–20 h bacterial culture using a DNA sorb-B kit (FBSI, Russia) according to the manufacturer's instructions. Amplification of the 16S rRNA gene was used with the following primers: 27f 5′-AGAGTTTGATCMTGGCTCAG-3′ and 1490r 5′-TACGGYTACCTTGTTACGACTT-3′^11^. Amplicons were visualized in the 1% agarose gel with ethidium bromide. The target products of ~ 1500 bp in length were extracted from agarose gel and purified via an agarose gel DNA extraction kit (Evrogen). Sanger sequencing was performed at the GENOME Center for Collective Use (Moscow, Russia). 16S rRNA gene consensus was assembled manually using the Unipro UGENE (v39.0) software^12^. The obtained consensus was searched against the EzTaxon server database^13^.

### Antibiotic susceptibility test of *E. marmotae* M-12

The susceptibility of *E. marmotae* M-12 to antibiotics was determined by the standard disc-diffusion method. Testing was performed following EUCAST 2022 guidance on the Mueller–Hinton agar (Himedia). The following discs with antibiotics were used (20 antibiotics in total): amoxicillin-clavulanic acid (20–10 µg), gentamicin (10 µg), trimetoprim-sulfametoxazol (1.25–23.75 µg) (NICF, Russia); aztreonam (30 µg), piperacillin (30 µg), piperacillin-tazobactam (100–10 µg), amikacin (30 µg), tobramycin (10 µg), ticarcillin-clavulanic acid (75–10 µg), ticarcillin (75 µg) (Bioanalyse, Turkey); ampicillin (10 µg), meropenem (10 µg), imipenem (10 µg), cefotaxime (5 µg), cefepime (30 µg), ceftazidime (10 µg), ciprofloxacin (5 µg), levofloxacin (5 µg), chloramphenicol (30 µg), trimethoprim (5 µg) (HiMedia, India).

### Detection of virulence factors expression

The expression of the following virulence factors was tested for *E. marmotae* M-12: capsule, hemolytic activity, and adhesion to human erythrocytes. Capsule was detected using Capsule Stain-Kit (HiMedia, India) by the instruction. The test was carried out with an overnight culture previously grown at 37 °C on Nutrient agar. *Klebsiella pneumoniae* ML-9 was used as the reference strain for capsule production (the strain from our local collection).

Hemolytic activity was evaluated onto the Columbian blood base agar (HiMedia, India) with 5% (vol/vol) mechanically defibrinated O-group human blood. Human blood was kindly provided by a volunteer with his written consent. All experiments were performed in accordance with relevant guidelines and regulations. The *E. marmotae* M-12 strain was incubated at 37 °C for 24 and 48 h. Alpha-hemolysis was interpreted as an absence of a clear lysis zone and the appearance of a green color around the colonies, beta-hemolysis was characterized as the presence of a clear lysis zone of red blood cells and gamma-hemolysis was considered as no lysis zone and absence of color changing^14^. *E. coli* X-1 Blue and *Staphylococcus aureus subsp. aureus Rosenbach* (ATCC 6538P) were used as negative (gamma-hemolysis) and positive (beta-hemolysis) control, respectively.

Adhesive properties were discovered through the method as described by Lenchenko et al.^15^. Mechanically defibrinated human blood of the O-group received from the same volunteer was used. Erythrocytes were obtained by washing defibrinated whole human blood in phosphate-buffered saline solution (PBS) (HiMedia, India). Defibrinated whole human blood was mixed with PBS pH 7.2 in a ratio of 1:1 and centrifuged at 1000 rpm for 10 min. Then the supernatant was discarded. Washing was repeated 2 times. The erythrocytes suspension (with PBS) was used in a concentration of 1 × 10^8^ cells/ml. Bacterial cell suspension with a density of 0.5 McFarland (~ 1.5 × 10^8^ microbial bodies per ml) was prepared from overnight cultures. Erythrocyte and bacterial suspensions were mixed in a 1:1 ratio (1 ml per 1 ml) and incubated at 37 °C for 30 min in a shaker incubator with shaking at 200 rpm. After incubation bacterial-erythrocytes suspension was washed three times with PBS pH 7.2 to wash out non-adhesion bacterial cells. Smears were prepared and stained with Giemsa stain solution. Adherent cells were counted through light microscopy. The following values were calculated: Average adhesion index (AAI)—the average number of microorganisms attached to the surface of a single red blood cell, all erythrocytes were counted in 5 fields of view, but not less than 50 erythrocytes; Adhesion coefficient (AC)—the percentage of red blood cells harboring bacteria on the surface; Microorganism adhesion index (AI) known as the ratio of AAI and AC. The following AI values were considered as: 1.00–1.75—non-adhesive; 1.75–2.49—low adhesive; 2.50–3.99—medium adhesive; > 4.00—high adhesive.

Biofilm production was detected via microtiter plate assay^16^. Briefly, bacterial suspension 0.5 McFarland is prepared in Mueller–Hinton Broth (MHB) with 1% glucose. Received bacterial suspension is 20-fold (1/20) diluted of MHB with 1% glucose. 180 μl of MHB with 1% glucose and 20 μl of bacterial suspensions are inoculated into 96-well flat-bottomed sterile polystyrene microplate. Microplates are incubated at 24 h at 37 °C. After incubation planktonic cells in wells are washed twice with phosphate-buffered saline (PBS) (pH 7.2) and wells are dried at 60 °C for 1 h. Biofilms formed on the walls of wells of microplate are stained with 150 μl of safranine for 15 min. Safranine-stained wells of microplates are washed twice with PBS. After drying in the air of the microplate, dye of biofilm walls of the microplate is resolubilized by 150 μl of 95% ethanol. Microplate is measured spectrophotometrically at 620 nm by a microplate reader. The studies are repeated in triplicates. Uninoculated wells containing sterile MHB supplemented with 1% are used as negative control. The negative control absorbance values (ODc) are used to identify whether biofilm formation of isolates exists or not. The wells of isolates of which OD values are higher than blank wells are considered to be biofilm producers.

### Cytotoxic assay

The cytotoxic assay was performed following dos Santos et al.^17^. HEp-2 cells were grown on a 96-well tissue culture plate with Dulbecco’s modified Eagle medium (DMEM) (Pan-Eco, Moscow, Russia) supplemented with 10% fetal bovine serum (FBS) (BioSera, USA) at 37 °C, 5% CO_2_. Bacterial cultures were incubated in 5 ml of double-strength tryptone soya broth TSB (HiMedia, India) at 37 °C for 18–24 h with shaking at 200 rpm. Overnight grown cultures were centrifuged at 10,000 × g at 4 °C for 2 min. The supernatants were filtered with a cellulose acetate filter (0.22 µm pore size). The filtrates were refrigerated for immediate use.

Wells with HEp-2 cell monolayers containing 200 µl DMEM with 2% FBS without antibiotics were inoculated with 40 µl and 20 µl of sterile filtrates to reach 1:5 and 1:10 final dilutions respectively. Plates were incubated at 37 °C in a 5% CO_2_ atmosphere. Cytotoxic effect was detected at 2, 24, and 48 h with inverted light microscopy. The cytotoxic positive effect was considered as disorganization of the cell monolayer, rounding, and shrinking of cells compared to cells untreated by filtrate. Experiments were repeated 3 times.

### Invasion and adhesion of *E. marmotae* M-12 to the HEp-2 cells

The methods of Cookson and Woodward^18^ were used with some modifications mentioned further. Bacterial cultures of *E. marmotae* M-12, *E. coli* XL-1 Blue, and *Salmonella enterica* subsp. *enterica* NCTC 5765 were previously grown in Nutrient broth for 3–4 h at 37 °C with shaking at 180 rpm to reach the logarithmic growth phase (OD = 0.5–0.7). Bacterial cells were washed with PBS three times and were stored in 15% glycerine (Sigma-Aldrich, USA) at − 80 °C until used. Colony-forming units (CFU) were determined via the standard plate count method (SPC).

The HEp-2 cells were cultivated directly in 24 well-tissue culture plates with DMEM medium (supplemented with 10% fetal bovine serum (FBS) (BioSera, USA) in a 5% CO_2_ atmosphere at 37 °C. Cells were used in 2 × 10^5^ concentration. Before use, cells were washed twice in PBS.

The multiplicity of infection (MOI) of all bacterial cultures was equal to approximately 200 units.

For the adhesion and invasion assay, the infected monolayers of HEp-2 cells with 1 ml of each bacterial inoculum were incubated for 3 h at 37 °C in a 5% CO_2_ atmosphere. The inocula were discarded after incubation and each well was immediately washed three times with PBS. Cells were disrupted after washing by the addition of PBS with 1% (v/v) Triton X-100 (Sigma, USA) into each well and further incubation for 5 min. The CFU was quantified via the SPC method.

For the invasion assay, the cell monolayers were additionally incubated for 2 h at 37 °C in a 5% CO_2_ atmosphere with DMEM supplemented with 100 µg/ml of gentamicin (Sigma, USA). After incubation with gentamicin, the cells were washed thrice with PBS and were disrupted by the addition of PBS with 1% (v/v) Triton X-100 (Sigma, USA) into each well and incubation for 5 min. The CFU of viable intracellular bacteria were quantified via the SPC.

*E. coli* XL-1 Blue and *Salmonella enterica* subsp. *enterica* NCTC 5765 were used as negative and positive control cultures respectively.

### Median lethal dose (LD_50_) assessment on mouse model

All procedures involving animals were approved by the Ethics Committee of the Federal Research Center for Virology and Microbiology (certificate No: IRECAS_01). All experiments were performed in accordance with relevant guidelines and regulations.

*Escherichia marmotae* M-12 culture was used in the exponential growth phase as mentioned above.

A total number of 60 mice (outbred mouse strain) of both sex with ages 6–8 weeks old and weight of 12–18 g were adapted for one week before starting the experiment. They were fed on ad libitum commercial assorted pellets and fresh clean water. 8 groups of 6 mice for the experiment and 2 groups of 6 mice for control were used.

The intraperitoneal, intranasal, and intravenous administration were utilized for LD50 assessment. The following inoculum doses were used for intraperitoneal and intranasal administration: 1 × 10^10^, 1 × 10^9^, and 1 × 10^8^ CFU/dose. For the intravenous injection, 1 × 10^5^ and 1 × 10^6^ CFU/dose were used.

### Genome sequencing, assembly and annotation of E. marmotae M-12

Overnight *E. marmotae* M-12 culture grown on Nutrient agar was used for DNA isolation. DNA was isolated with the QIAamp DNA Mini Kit (QIAGEN, Germany). Sequencing was performed by Geoanalytics (Russia) using Illumina HiSeq1500 with 150-bp paired-end reads and coverage of 100×.

All bioinformatic procedures were performed on Galaxy^19^. Read quality assessment was made with the FastQC v0.11.9 tool (http://www.bioinformatics.babraham.ac.uk/projects/fastqc). Reads trimming was performed via Trimmomatic v0.38 with default parameters^20^. The *E. marmotae* M-12 genome assembly was carried out with Unicycler v0.4.8^21^. Contigs shorter than 100 bp in length were excluded from the *E. marmotae* M-12 assembly. The annotation of the *E. marmotae* M-12 genome was carried out with Prokka v1.14.6 tool using standard parameters^22^.

### Phylogenomic analysis

The phylogenetic tree was reconstructed with the REALPHY v1.13 online tool^23^. *E. marmotae* HT073016 type strain was used as the reference. 51 genons not repeated or had not an empty file out of 62 *E. marmotae* genomes downloaded from NCBI GenBank were used (Table S1). The phylogenetic tree was visualized with the Interactive Tree Of Life (iTOL) version 6.6 online tool^24^. The midpoint rooting approach was utilized to root the final phylogenetic tree.

### Comparative analysis of *E. marmotae* virulence factors

Comparative analysis was performed on the Galaxy server^19^. All genomes of *E. marmotae* from NCBI GenBank were used. Also, some genomes repeated or had an empty file, these genomes were not used in analysis. Thus, 51 out of 62 published genomes of *E. marmotae* and strain M-12 were used for comparative analysis of virulence factors (VFs) (Table S1). Genomes were annotated using the Prokka tool v1.14.6^22^.

Protein sequences of the Virulence Factor Database (VFDB) core dataset (set A) were used (v2022.04.05)^25^. Blastp tool was applied for virulence factors searching with the following parameters: E-value ≤ 1e−30, bit score ≥ 100, percentage of identity ≥ 60%, and minimum query coverage ≥ 85^26,27^.

## Results

### Isolation and identification of *E. marmotae* M-12

The M-12 isolate was obtained from the lungs of a male bank vole (*Myodes glareolus*). Culture M-12 was stored in NB with 15% glycerol at − 80 °C.

Microscopy investigation revealed a single Gram-negative coccobacillus in M-12 isolate. Catalase-positive and oxidase-negative reactions were observed. Isolate M-12 exhibited a motility phenotype at 37 °C. M-12 had pink colonies and a negative H_2_S reaction on XLD agar. The ENTEROtest 24N test was used for the biochemical characterization of M-12. The following biochemical reactions were positive: lysine, ß-galactosidase, melibiose, trehalose, mannitol, and ß-glucuronidase. The negative ones were: urease, arginine, ornithine, hydrogen sulfide, Simmons citrate, malonate, salicin, sorbitol, cellobiose, lactose, dulcin, adonitol, arabitol, sucrose, inositol, raffinose, esculin, ß-xylosidase. The biochemical profile of the M-12 isolate was identified as “*Escherichia coli* inactive” with 61.53% of identity.

We performed further identification via 16S rRNA gene sequencing with 27f and 1492r universal primers. The assembled consensus of the 16S rRNA gene was 649 bp for the M-12 isolate. The highest sequence identity of M-12 consensus was 100% with the 16S rRNA gene sequence of the *E. marmotae* HT073016 (T).

### Antimicrobial susceptibility profile of *E. marmotae* M-12

Antimicrobial susceptibility was tested through a disc-diffusion method according to the EUCAST 2022 guidance. In total antimicrobial susceptibility to 20 antibiotics was determined. Among the beta-lactams and aminoglycosides groups resistance was detected to ampicillin, amoxiclav, and tobramycin. *E. marmotae* M-12 was susceptible to ticarcillin, ticarcillin-clavulanate, piperacillin, piperacillin-tazobactam, aztreonam, meropenem, imipenem, cefotaxime, ceftazidime, cefepime, gentamicin, amikacin, ciprofloxacin, levofloxacin, chloramphenicol, trimethoprim, trimetoprim-sulfametoxazol (Table 1).

**Table 1:** Results of antimicrobial susceptibility testing for *E. marmotae* M-12.

Antibiotic class	Antibiotics	Zone, mm	Interpretation
Beta-lactams	Ampicillin	0	R
	Amoxiclav	13	R
	Ticarcillin	24	S
	Ticarcillin-clavulanate	26	S
	Piperacillin	24	S
	Piperacillin-tazobactam	23	S
	Aztreonam	32	S
	Meropenem	30	S
	Imipenem	28	S
	Cefotaxime	30	S
	Ceftazidime	28	S
	Cefepime	32	S
Aminoglycosides	Gentamicin	19	S
	Amikacin	20	S
	Tobramycin	13	R
Fluoroquinolones	Ciprofloxacin	29	S
	Levofloxacin	28	S
Other	Chloramphenicol	27	S
	Trimethoprim	32	S
	Trimethoprim-sulfamethoxazole	28	S

### Secreted virulence factors of *E. marmotae* M-12

**Figure 1:**
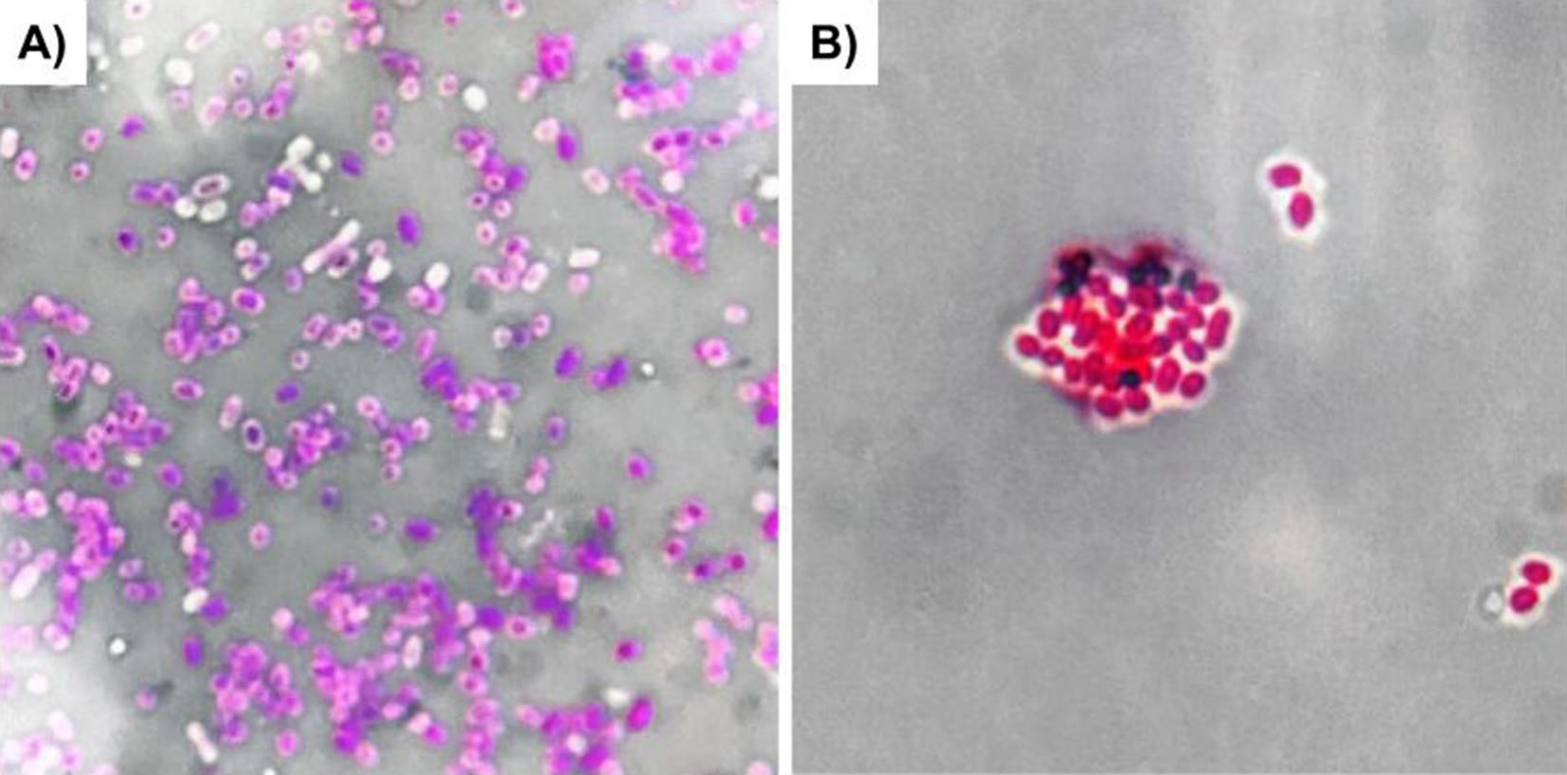
Light microscopy of capsules in the dark background (× 1800): (**A**) *E. marmotae* M-12 stained with gentian violet; (**B**) strain *K. pneumoniae* ML-9 stained with fuchsine.

Microscopy in the dark background was carried out to detect the capsule of *E. marmotae* M-12. *K. pneumoniae* ML-9 was used as a control. Strain *E. marmotae* M-12 exhibited a capsule-positive phenotype (Fig. 1A). However, visually *E. marmotae* M-12 isolate had a smaller capsule than *K. pneumoniae* ML-9 (Fig. 1B).

Hemolytic activity of *E. marmotae* M-12 was not observed on blood agar with 5% O-group human blood at 37 °C for 24–48 h (Gamma-hemolysis).

**Figure 2:**
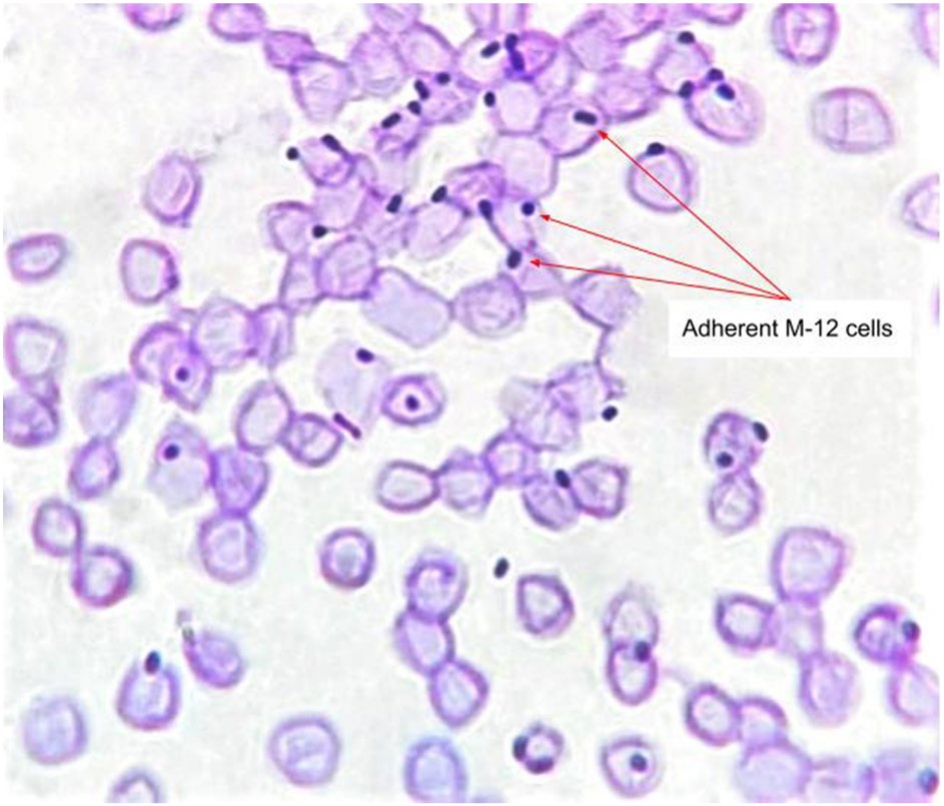
Adhesion of *E. marmotae* M-12 bacterial cells to human erythrocytes (× 1800). Stained with Giemsa stain solution.

Adhesion of *E. marmotae* M-12 cells to the human erythrocytes (O-group) was discovered through the method described by Lenchenko et al.^15^ *E. marmotae* M-12 strain had 5.6 units of microorganism adhesion index (AI). This AI level indicates high adhesive properties of *E. marmotae* M-12 to O-group human erythrocytes (Fig. 2).

### Cytotoxic assay

Detection of the cytotoxic effect of *E. marmotae* M-12 on HEp-2 cells was performed using inverted light microscopy. Cytotoxic effect of *E. marmotae* M-12 to HEp-2 cells was not detected after 2, 24, and 48 h of incubation: disorganization of the cell monolayer as well as rounding and shrinking of cells was not observed.

### Adhesion and invasion activity of *E. marmotae* M-12 to HEp-2 cells

**Figure 3:**
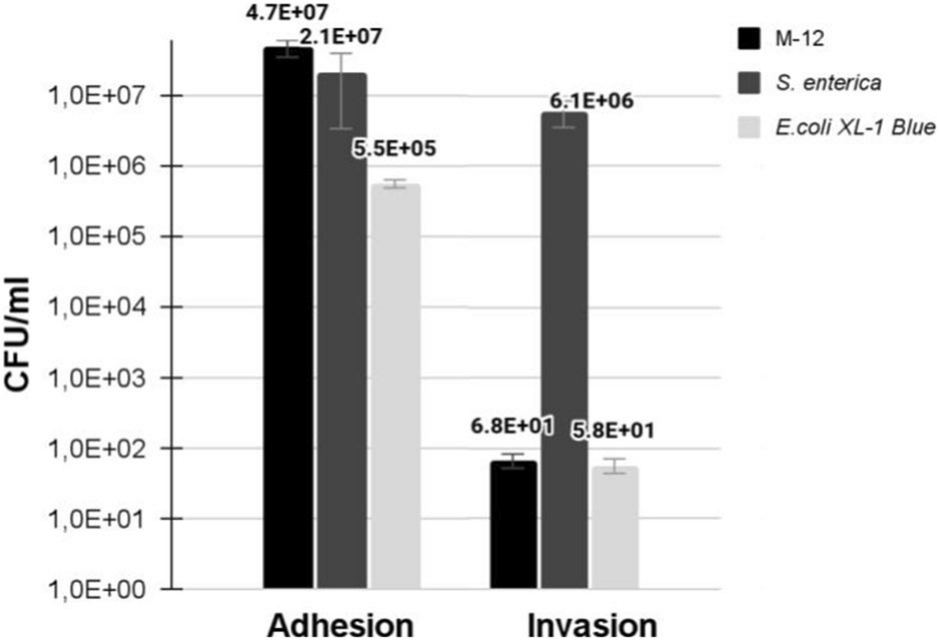
Adhesion and invasion activity of HEp-2 cells following infection by *E. marmotae* M-12, *S. enterica subsp. enterica* NCTC 5765, and *E. coli* XL-1 Blue. Statistical processing of data was performed via Newman–Keuls test. Adhesion activity between all strains had statistically significant differences (p ≤ 0.05). Invasion activity of *E. coli* XL-1 Blue and *E. marmotae* M-12 in comparison with the control *S. enterica* NCTC 5765 had statistically significant differences (p ≤ 0.01), while *E. coli* XL-1 Blue and *E. marmotae* M-12 did not have statistically significant differences. Results represented as means ± SD from at least three independent experiments in duplicate.

Adhesion and invasion activity of *E. marmotae* M-12 to HEp-2 cells was observed (Fig. 3). *S. enterica* subsp. *enterica* NCTC 5765 and *E. coli* XL-1 Blue were used as controls. Determination of the adhesive and invasive activities was conducted after 3 h of HEp-2 incubation with bacterial cultures. Adhesive activity of *E. marmotae* M-12 was 4.7 × 10^7^ CFU/ml while invasive activity was 68 CFU/ml. Statistical processing of data was performed via Newman–Keuls method.

Adhesion of *E. marmotae* M-12 was higher than in *S. enterica* NCTC 5765 by 2.24 times and higher than in *E.* coli XL-1 Blue by 85.45 times. Statistically significant differences were observed between all groups (p ≤ 0.05). Statistically significant differences were also found when comparing the following groups: *E. marmotae* M-12*—E. coli* XL-1 Blue, and *E. marmotae* M-12—*S. enterica* NCTC 5765 (p ≤ 0.01).

The invasion activity of E. *marmotae* M-12 into HEp-2 cells was 68 CFU/ml and was similar in *E. coli* XL-1 Blue (58 CFU/ml). The invasion activity of *S. enterica* NCTC 5765 was 6.1 × 10^6^ CFU/ml. Therefore, the invasive activity of *E. marmotae* M-12 was higher than in *E. coli* XL-1 by more than 1.17 times and less than in *S. enterica* NCTC 5765 by more than 105,000 times. Statistically significant differences were observed between the *E. coli* XL-1 Blue—*S. enterica* NCTC 5765 group and *E. marmotae* M-12—*S. enterica* NCTC 5765 group (p ≤ 0.01). *E. coli* XL-1 Blue and *E. marmotae* M-12 did not have statistically significant differences among themselves. Thus, strain *E. marmotae* M-12 are non-invasive to into HEp-2 cells, because invasive values of *E. marmotae* M-12 and *E. coli* XL-1 Blue (negative control) were not statistically significant differences.

### Biofilm production assay of *E. marmotae* M-12

Biofilm production was detected via microtiter plate assay method. *E. marmotae M-12* strain did not produce biofilm in wells of microtiter plate. Average OD of test wells was 0.041. ODc = 0.041 had negative control wells. Thus, if OD ≤ ODc strain *E. marmotae M-12* has no biofilm production^16^.

### Median lethal dose (LD_50_) of *E. marmotae* M-12 for mice

The results of LD_50_ for *E. marmotae* M-12 in mice after injection of different bacterial doses to intraperitoneal, intranasal, and intravenous are shown in Table 2. Alive and dead mice were counted for 7 days. Death of all animals in the 1 group (intraperitoneal administration with 1 × 10^10^ dose) was observed on the first day after infection. Other animals in all groups were alive throughout the experiment.

**Table 2:** LD_50_ assessment of *E. marmotae* M-12 in mouse infection model.

Group	Route of administration	CFU/dose	Alive	Dead	Percentage
1	Intraperitoneal	1 × 10^10^	0	6	100
2		1 × 10^9^	6	0	0
3		1 × 10^8^	6	0	0
4	Intranasal	1 × 10^10^	6	0	0
5		1 × 10^9^	6	0	0
6		1 × 10^8^	6	0	0
7	Intravenous	1 × 10^5^	6	0	0
8		1 × 10^6^	6	0	0
9	Intraperitoneal	PBS	6	0	0
10	Intranasal	PBS	6	0	0

### Assembly and annotation genome of *E. marmotae* M-12

The assembly *E. marmotae* M-12 genome consisted of 139 contigs with a total length of 4.9 Mb. The average read coverage was 607×, GC% was 50.37. The genome of *E. marmotae* M-12 was deposited in NCBI GenBank under accession GCA_029102925.1.

The 4957 CDS, 76 tRNA, and 4 rRNA features were annotated in the M-12 chromosome. In total, 4957 protein features were predicted, among them 4239 proteins with functional assignments and 718 hypothetical proteins.

### Phylogenomic analysis of *E. marmotae* M-12

**Figure 4:**
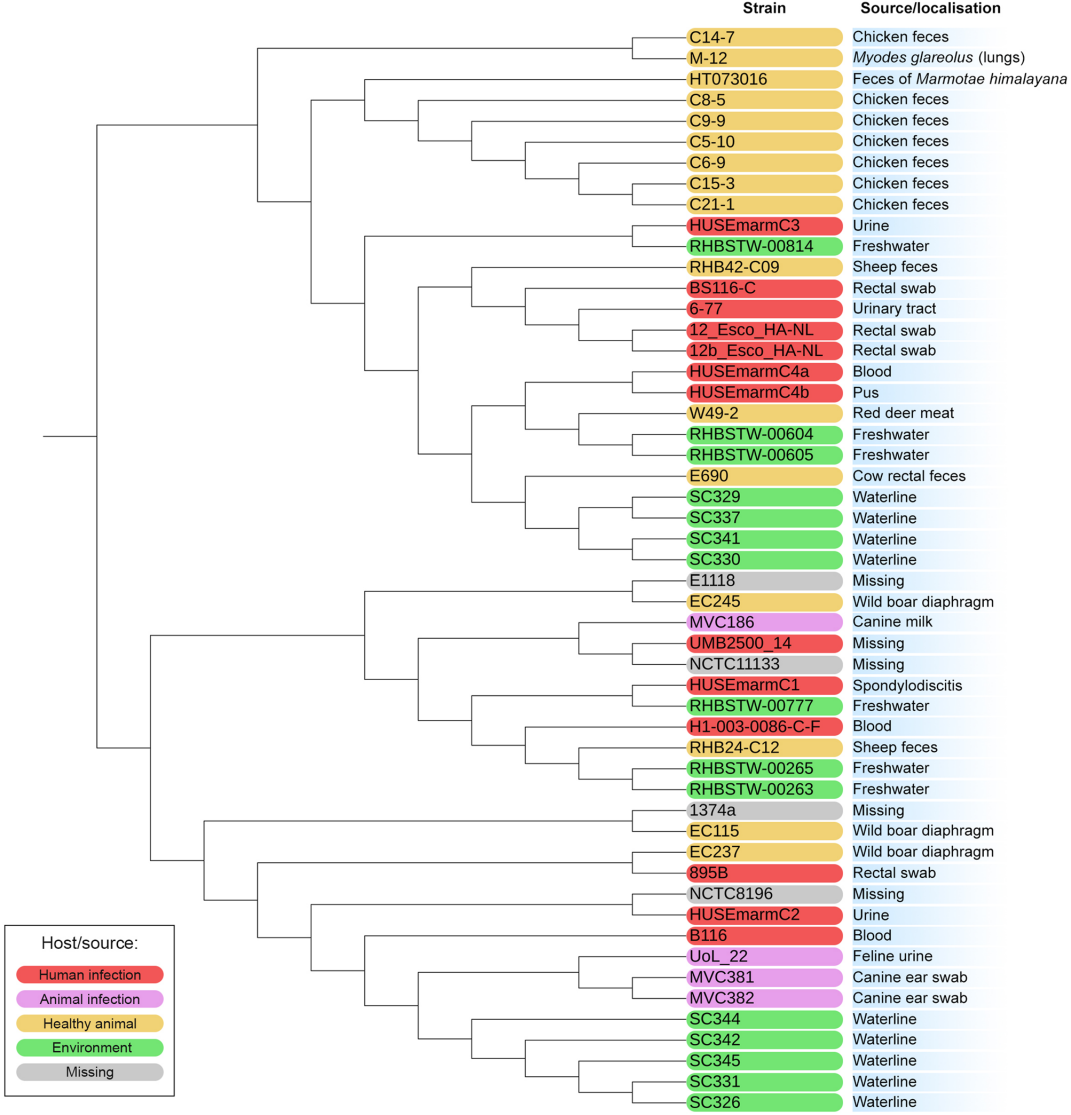
Phylogenetic tree of *E. marmotae* reconstructed with REALPHY 1.13.

A whole genome-based phylogenetic analysis of *E. marmotae* M-12 was carried out via REALPHY 1.13. *The E. marmotae* M-12 strain was phylogenetically related to the *E. marmotae* C14-7 and formed a sister group with the latter. Notably, *E. marmotae* C14-7 was isolated from the fecal of healthy chicken (*Gallus gallus*) in the United Kingdom in 2018. In general, phylogenomic analyses of *E. marmotae* showed dispersed phylogenetic structure among isolates of different origins (Fig. 4).

### Virulome and resistome analysis of *E. marmotae* M-12

The *E*. *marmotae* M-12 had 160 homologous proteins of VFs in different groups such as biofilm and capsule formation, siderophores, virulence regulation, adherence, nutritional/metabolic factors, type II and VI secretion systems (T2SS, T6SS), invasion, and motility factors (Table S2).

The homolog of the LuxS of *Vibrio cholerae* O1 biovar El Tor in the biofilm category was detected in the M-12 with an identity of 72.5%. LuxS is widely conserved among gram-negative and gram-positive bacteria and is associated with quorum sensing and biofilm formation^28^.

12 homolog proteins from the capsule category of the *Klebsiella pneumoniae* were found: KP1_RS17280, KP1_RS17295, KP1_RS17305, KP1_RS17340, RfbK1, GndA, RcsA, RcsB, Ugd, WcaJ, Gmd, GalF. Capsule assists in evading the host immune system by protecting bacteria from opsonophagocytosis and serum killing^29^.

12 homolog proteins needed for enterobactin synthesis (Fes, EntA, EntB, EntC, EntD, EntE, EntF, EntS, FepA, EntB, EntC, EntD, EntE, EntG) from the *E. coli* were found with the identity of 79–99.6% in siderophore category^30^.

Also, all the PhoPQ homologs of VFs in the regulator category from the *Salmonella enterica* were detected with PhoP 94% and PhoQ 86% identity. Additionally, two regulatory proteins RpoS and Fur of *S. enterica* with an identity of 99% were found. PhoPQ proteins control the expression of more than 40 genes as well as are required for intracellular survival, cationic antimicrobial peptides (CAMPs) resistance, and stimulation of cytokine secretion^31^. Also, PhoPQ regulon and RpoS protein provide acid resistance of *Salmonella*^32,33^. Fur protein, in turn, represses the expression of iron-regulated genes^34^.

In the adherence category, curli fimbriae-associated proteins from *E. coli* were found in the M-12 genome with identity 92–98% (CgsG, CgsF, CgsE, CgsD, CgsB, CgsA, CgsC). The type I fimbriae proteins from *E. coli* with 88.5–98% identity were also discovered (FimB, FimE, FimA, FimI, FimC, FimD, FimF, FimG, FimH). Production of extracellular curli proteins primarily participates in adhesion and biofilm formation^35^, while type I fimbriae proteins are associated with urinary tract infection by extraintestinal pathogenic *E. coli* (ExPEC)^36^.

In the nutritional/metabolic factor category heme uptake proteins group consisting of 8 proteins were found from *E. coli* with 93.5–98% identity (ChuS, ChuA, ChuT, ChuW, ChuX, ChuY, ChuU, ChuV). The allantoin utilization system of *K. pneumoniae* was found, which includes 6 proteins with an identity of 73–91% (AllS, AllA, AllR, AllB, AllC, AllD). Heme uptake proteins have been shown to transport heme using proteoliposomes and hydrolyze ATP^37^. An allantoin utilization operon has been providing a nitrogen source to increase virulence in *K. pneumoniae* and is associated with hypervirulent strains that cause pyogenic liver abscesses^38^.

In the motility category flagella complex composed of 7 subunits from the *Yersinia enterocolitica* with identity 68–91% were detected (CheZ, CheY, CheB, CheR, CheD, CheW, CheA). Fli, Flg, and Fin flagella complexes were not completed and contained separate proteins. Flagella complex proteins are associated with intracellular invasion and biofilm formation.

10 out of 13 proteins of the type II secretion system (T2SS) from the *Shigella dysenteriae* with 90–98% identity were found in the M-12 isolate. Outer membrane protein (GspD), leader peptidase (GspO), and inner membrane protein (GspM) were absent in *E. marmorae* M-12.

Among invasive VFs, the OmpA porin with an identity of 96%, as well as IbeA, IbeB, and IbeC with 95–99% identity, were detected from *E. coli*. These VFs are associated with the pathogenicity of ExPEC. OmpA porin and Ibe proteins cause intracellular migration in neonatal meningitis as well as intracellular invasion and migration in cystitis^39–42^.

*Escherichia marmotae* M-12 had 39 AMR factors in the genome (Table S3). Among these, we discovered beta-lactamase: *ampC* and *ampH* to resistance to penicillin-like and cephalosporin-class antibiotics. Also, some genes conferring resistance to beta-lactams were found: *mdtE*, *mdtF*, *gadX*, *H-NS*, *marA*, *tolC*, *acrB*, *acrA.*

*AcrD*, *cpxA*, *baeS*, *baeR*, *tolC*, *kdpE* genes conferring resistance to aminoglycoside were found. *AcrD* is an aminoglycoside efflux pump expressed in *E. coli*. Its expression can be induced by indole and is regulated by *baeRS* and *cpxAR*.

### Comparative virulome analysis of *E. marmotae*

51 *E. marmotae* genomes were used for comparative VFs analysis. Search for VFs was conducted against the VFDB core dataset (set A) via the Blastp tool. The following VF categories were assessed for comparative analysis: siderophore, adherence, regulator factors, nutritional/metabolic factors, bacterial secretion systems, and invasion factors (Table S2).

All strains studied had enterobactin-associated proteins with 79–99.6% identity from the *E. coli* CFT073 (Fes, EntA, EntB, EntC, EntD, EntE, EntF, EntS, FepA, EntB, EntC, EntD, EntE, EntG). Only the *E. marmotae* UoL_22, MVC382, MVC381 strains had complete protein set for yersiniabactin synthesis with an identity of 99–100% from the *Yersinia pestis* CO92 (YbtT, YbtE, YbtU, YbtA, YbtQ, YbtX, YbtP, YbtS, Irp1, Irp2, FuyA). Only the *E. marmotae* 6-77 strain had salmochelin-associated proteins with 98–100% identity from *E. coli* CFT073 (IroN, IroE, IroD, IroC, IroB).

All *E. marmotae* strains had all 7 curli fimbriae proteins from *E. coli* O25b:H4-ST131 with 92–98% identity. The total set of type I fimbriae proteins from *E. coli* CFT073 with 88.5–98% identity was detected in almost all strains except *E. marmotae* C8-5, C9-9, SC345, SC344, SC342, SC331, SC326.

PhoQ, PhoP, RpoS, and Fur regulatory proteins from *S. enterica* subsp. *enterica* serovar Typhimurium LT2 were found in all *E. marmotae* strains with an identity of 86, 94, 98.6, and 99% respectively.

A full set of 8 proteins of the heme uptake group from *E. coli* CFT073 with 93.5–98% identity were found in all strains. A full set of allantoin utilization factors were also detected in all strains except HT073016 (T) and C14-7 strains.

The Type II secretion system (T2SS) and Type VI secretion system (T6SS) were not completed among all strains. The following strains did not have any T2SS proteins: C21-1, C8-5, C15-3, C6-9, SC345, SC344, SC342, SC331, SC326. Type III secretion system (T3SS) proteins were found only in the HT073016 type strain with an identity of 60–94% from *Shigella flexneri* 2a301. However, the following T3SS proteins had an identity lower than 60% (46–52%): MxiG, MxiK, MxiN, and Spa13. Additionally, only the HT073016 (T) strain had effector proteins IpaH7,8 and IpaH9,8 with an identity of 62% and 90% respectively.

From the invasion VFs category, OmpA from *E. coli* O18:K1:H7 RS218, IbeB, and IbeC from *E. coli* O45:K1:H7 S88 with 95%, 95%, and 98% identity respectively were discovered in all strains. IbeA protein was found in the following strains: H1-003-0086-C-F, 895B, HUSEmarmC2, HUSEmarmC1, MVC186, EC115, EC237, RHB42-C09, RHB24-C12, C21-1, C14-7, C5-10, C15-3, C6-9, C9-9, 1374a, SC345, SC344, SC342, SC341, SC337, SC331, SC329, SC326, SC330, RHBSTW-00777, RHBSTW-00265, RHBSTW-00263. The AslA protein needed for invasion in brain microvascular endothelial cells (BMECs) was found in all strains with an identity of 96% from *E. coli* O18:K1:H7 RS218 except C8-5 and E1118 strains.

The full protein set of the capsule K1 synthesis from *E. coli* O45:K1:H7 S88 was detected only in the H1-003-0086-C-F strain with 95–100% identity. Notably, strains isolated from sick humans, animals, and the environment had only Kps proteins of the capsule K1 synthesis pathway. Kps proteins had an identity percentage of 70–98% with *E. coli* O45:K1:H7 S88. Strains isolated from healthy wild and farm animals did not have any proteins related to capsule K1.

## Discussion

*Escherichia marmotae* are Gram-negative bacteria of *the Enterobacterales* order. This bacterium was first isolated from the Himalayan marmot (*Marmota himalayana*) and described in 2015^2^. After description *E. marmotae* strains were isolated from wild animals: the feces of an *Alpine marmot*^3^ and *Marmota himalayana*^6^; from farm animals: cow rectal feces^4^, fresh fecal samples of healthy hens^5^; from sick companion animals (canines and felines); from freshwater samples from downstream wastewater treatment plants, and waterline^43^. Apparently, *E. marmotae* has a wide distribution, particularly in animals and water environments which may serve as the reservoirs for these bacteria. We isolated *E. marmotae* M-12 from the lungs of bank vole (*Myodes glareolus*). The latter also supports the assumption about animal reservoirs of this bacterium species.

It has been also shown that some *E. marmotae* strains may be pathogenic for humans. In 2022 the first case of *E. marmotae* infection in humans was observed in Norway. There were 4 extraintestinal infection cases in which *E. marmotae* caused sepsis of unknown origin, postoperative sepsis, and upper urinary tract infection^7^. GenBank also has information about other clinical *E. marmotae* strains that were isolated from a urinary tract infection, blood samples, and rectal swabs. Mentioned above indicates that *E. marmotae* is truly pathogenic for humans and can cause severe infections. We performed a phylogenomic analysis of *E. marmotae* to elucidate a potential correlation between sources and the phylogeny of *E. marmotae* isolates. Interestingly, the phylogenomic analyses showed pathogenic isolates from humans dispersed among isolates with environmental and animal origin, indicating a common virulence profile among different lineages. The same findings were observed by Sivertsen et al.^7^.

To investigate virulence factors and assess potential pathogenicity we performed comparative virulome analysis among *E. marmotae* strains with different origins: healthy or sick humans and animals, and environment. A high number of virulence-related proteins were associated with the *Escherichia* genus which corresponds well with previous investigation^7^. The following factors were observed among all *E. marmotae* strains: enterobactin-associated proteins, heme uptake proteins, capsule, and biofilm synthesis proteins, curli and type I fimbriae, motility machinery proteins, and OmpA porin. Despite the presence of biofilm formation VFs in the genome, *E. marmotae* M-12 did not produce biofilm in vitro. Notably, OmpA porin is known to confer adhesion of *E. coli* cells to human brain microvascular endothelial cells and plays a major role in meningitis pathogenesis in humans^44^. Other factors mentioned above are responsible for adhesion, survival, and dissemination in a host^45–47^.

Interestingly, strains from healthy and sick humans and animals had differences in virulomes among each group (healthy and sick). For instance, proteins associated with capsule K1 synthesis and secretion were found only in *E. marmotae* isolated from sick humans and animals as well as in some environmental strains. The K1 capsule is an important virulence factor contributing to *E. coli* meningitis pathogenesis. It was shown that the K1 capsule allows *E. coli* to internalize itself into human brain microvascular endothelial cells (BMEC)^48^. Other disease manifestations caused by *E. coli* K1 include urinary tract infections and sepsis^49,50^. We suggest that capsule K1 and OmpA are the major virulence factors contributing to *E. marmotae* infection. Possibly, K1 capsule and OmpA porin determine pathogenesis similarly to extraintestinal pathogenic *E. coli* (ExPEC)*,* for example, uropathogenic *E. coli* (UPEC) or neonatal meningitis *E. coli* (NMEC)^7,47^. It well corresponds with manifestations of *E. marmotae* infection cases described in Norway including sepsis and urinary tract infection^7^. Moreover, detected factors such as fimbriae, flagellum, siderophores, and heme uptake proteins may play additional roles in *E. marmotae* infectious process development. However, this hypothesis is based only on the comparative virulome analysis of *E. marmotae* strains and analysis of cases described in the literature. Further experimental research is necessary to support our assumptions.

We assessed the pathogenic potential of our *E. marmotae* M-12 isolate using culture-based methods as well as a mouse infection model. *E. marmotae* M-12 had a high adhesion level to human red blood cells and HEp-2 cells. High adhesion may be related to the presence of curli fimbriae genes as well as type I fimbriae genes observed in the M-12 isolate genome^51,52^. *E. marmotae* M-12 strain was not invasive in HEp-2 cells. Notably, *E. marmotae* M-12 and HT073016 strains had almost the same invasion activity in HEp-2 cells: 68 CFU for M-12 and 46 CFU for HT073016^6^. The invasion of gram-negative bacteria through the trigger mechanism is known to be mostly related to the type III secretion system (T3SS)^53–56^. Notably, only *E. marmotae* HT073016 had proteins of T3SS among all *E. marmotae* strains under consideration. Perhaps, the invasive phenotype of *E. marmotae* is not associated with the presence of T3SS and is rather related to the zipper mechanism^57^. However, further investigations are needed to evaluate the role of T3SS in *E. marmotae* invasion and virulence.

We used the mouse model to evaluate the virulence of *E. marmotae* M-12. Death of laboratory mice was detected only with infection dose 1 × 10^10^ CFU on intraperitoneal administration. Death in laboratory mice was probably due to toxic shock under high doses of bacterial cells. Based on our findings *E. marmotae* M-12 is not virulent for mice. However, *Marmota himalayana* and other *Rodentia* including mice may be a possible reservoir for *E. marmotae*, they may be naturally resistant to this causative agent^6^. Although, to date, there is no definite evidence of mice as a natural reservoir of *E. marmotae*.

Phenotypic resistance to beta-lactams and aminoglycosides was perhaps associated with *mdtE*, *mdtF*, *gadX*, *H-NS*, *marA*, *tolC*, *acrB*, *acrA*, and *acrD*, *cpxA*, *baeS*, *baeR*, *tolC*, *kdpE* genes, respectively, to be detected in the genome of *E. marmotae* M-12 strain.

In conclusion, our study reported the first case of *E. marmotae* isolation from the lungs of a bank vole (*Myodes glareolus*). Presumably, wild animals especially of *Rodentia* order may be a natural reservoir for *E. marmotae* as was suggested previously*. E. marmotae* M-12 reported in this work had a capsule-positive phenotype, high adhesion to human erythrocytes and HEp-2 cells as well as it was non-invasive into HEp-2 cells. *E. marmotae* M-12 had no hemolysis to human erythrocytes, cytotoxicity to HEp-2 cells, and was not virulent in a mouse model. Based on comparative genomic analysis we assume the K1 capsule and OmpA porin of *E. marmotae* may be the key virulence factors. Perhaps, the latter factors determine the pathogenesis of *E. marmotae* infection in a similar manner to extraintestinal pathogenic *E. coli* (ExPEC). However, this hypothesis is based only on the comparative virulome analysis of *E. marmotae* strains and analysis of cases described in the literature. Further experimental research is necessary to support our assumptions.

### Supplementary Information

Supplementary Table S1.

Supplementary Table S2.

Supplementary Table S3.

## Data Availability

Genome assembly of *Escherichia marmotae* M-12 is available in the GenBank repository: accession no. GCA_029102925.1.

## References

[1] Denamur E, Clermont O, Bonacorsi S, Gordon D (2021). The population genetics of pathogenic *Escherichia coli*. Nat. Rev. Microbiol..

[2] Liu S (2015). *Escherichia marmotae* sp. nov., isolated from faeces of Marmota himalayana. Int. J. Syst. Evol. Microbiol..

[3] De Witte C (2021). Presence of broad-spectrum beta-lactamase-producing enterobacteriaceae in zoo mammals. Microorganisms.

[4] Ocejo M, Tello M, Oporto B, Lavín JL, Hurtado A (2020). draft genome sequence of *Escherichia marmotae* E690, isolated from beef cattle. Microbiol. Resour. Announc..

[5] Thomson NM (2022). Remarkable genomic diversity among *Escherichia* isolates recovered from healthy chickens. PeerJ.

[6] Liu S (2019). Genomic and molecular characterisation of *Escherichia marmotae* from wild rodents in Qinghai-Tibet plateau as a potential pathogen. Sci. Rep..

[7] Sivertsen A (2022). *Escherichia marmotae*—a human pathogen easily misidentified as *Escherichia coli*. medRxiv.

[8] du Sert NP (2020). The arrive guidelines 2.0: Updated guidelines for reporting animal research. PLoS Biol..

[9] du-Sert NP (2020). Reporting animal research: Explanation and elaboration for the ARRIVE guidelines 2.0. PLoS Biol..

[10] Scudamore CL, Busk N, Vowell K (2014). A simplified necropsy technique for mice: Making the most of unscheduled deaths. Lab. Anim..

[11] Weisburg WG, Barns SM, Pelletier DA, Lane DJ (1991). 16S ribosomal DNA amplification for phylogenetic study. J. Bacteriol..

[12] Okonechnikov K (2012). Unipro UGENE: A unified bioinformatics toolkit. Bioinformatics.

[13] Yoon SH (2017). Introducing EzBioCloud: A taxonomically united database of 16S rRNA gene sequences and whole-genome assemblies. Int. J. Syst. Evol. Microbiol..

[14] Buxton, R. Blood Agar Plates and Hemolysis Protocols. *ASM.* 1–9. https://asm.org/getattachment/7ec0de2b-bb16-4f6e-ba07-2aea25a43e76/protocol-2885.pdf (2005).

[15] Lenchenko E, Blumenkrants D, Sachivkina N, Shadrova N, Ibragimova A (2020). Morphological and adhesive properties of Klebsiella pneumoniae biofilms. Vet. World.

[16] Kırmusaoğlu S, Kırmusaoğlu S (2019). The methods for detection of biofilm and screening antibiofilm activity of agents. Antimicrob. Antibiot. Resist. Antibiofilm Strateg. Activity Methods.

[17] Dos-Santos PA (2015). Adhesion, invasion, intracellular survival and cytotoxic activity of strains of *Aeromonas* spp. in HEp-2, Caco-2 and T-84 cell lines, Antonie van Leeuwenhoek. Int. J. Gener. Mol. Microbiol..

[18] Cookson AL, Woodward MJ (2003). The role of intimin in the adherence of enterohaemorrhagic *Escherichia coli* (EHEC) O157: H7 to HEp-2 tissue culture cells and to bovine gut explant tissues. Int. J. Med. Microbiol..

[19] Afgan E (2022). The Galaxy platform for accessible, reproducible and collaborative biomedical analyses: 2022 update. Nucleic Acids Res.

[20] Bolger AM, Lohse M, Usadel B (2014). Trimmomatic: A flexible trimmer for Illumina sequence data. Bioinformatics.

[21] Wick RR, Judd LM, Gorrie CL, Holt KE (2017). Unicycler: Resolving bacterial genome assemblies from short and long sequencing reads. PLoS Comput. Biol..

[22] Seemann T (2014). Prokka: Rapid prokaryotic genome annotation. Bioinformatics.

[23] Bertels F, Silander OK, Pachkov M, Rainey PB, Van Nimwegen E (2014). Automated reconstruction of whole-genome phylogenies from short-sequence reads. Mol. Biol. Evol..

[24] Letunic I, Bork P (2021). Interactive tree of life (iTOL) v5: An online tool for phylogenetic tree display and annotation. Nucleic Acids Res..

[25] Liu B, Zheng D, Zhou S, Chen L, Yang J (2022). VFDB 2022: A general classification scheme for bacterial virulence factors. Nucleic Acids Res..

[26] Cock PJA, Chilton JM, Grüning B, Johnson JE, Soranzo N (2015). NCBI BLAST+ integrated into galaxy. Gigascience.

[27] Camacho C (2009). BLAST+: Architecture and applications. BMC Bioinform..

[28] Chen X (2002). Structural identification of a bacterial quorum-sensing signal containing boron. Nature.

[29] Paczosa MK, Mecsas J (2016). *Klebsiella pneumoniae*: Going on the offense with a strong defense. Microbiol. Mol. Biol. Rev..

[30] Raymond KN, Dertz EA, Kim SS (2003). Enterobactin: An archetype for microbial iron transport. Proc. Natl. Acad. Sci. U. S. A..

[31] Ohl ME, Miller SI (2001). Salmonella: A model for bacterial pathogenesis. Annu. Rev. Med..

[32] Lucas RL, Lee CA (2000). Unravelling the mysteries of virulence gene regulation in *Salmonella typhimurium*. Mol. Microbiol..

[33] Fang FC (1992). The alternative sigma factor katF (rpoS) regulates *Salmonella virulence*. Proc. Natl. Acad. Sci..

[34] Tsolis RM, Baumler AJ, Stojiljkovic I, Heffron F (1995). Fur regulon of *Salmonella typhimurium*: Identification of new iron-regulated genes. J. Bacteriol..

[35] Barnhart MM, Chapman MR (2006). Curli biogenesis and function. Annu. Rev. Microbiol..

[36] Stærk K (2021). *Escherichia coli* type-1 fimbriae are critical to overcome initial bottlenecks of infection upon low-dose inoculation in a porcine model of cystitis. Microbiol. U. K..

[37] Richard KL, Kelley BR, Johnson JG (2019). Heme uptake and utilization by gram-negative bacterial pathogens. Front. Cell Infect. Microbiol..

[38] Chou HC (2004). Isolation of a chromosomal region of *Klebsiella pneumoniae* associated with allantoin metabolism and liver infection. Infect. Immun..

[39] Krishnan S, Prasadarao NV (2012). Outer membrane protein A and OprF: Versatile roles in Gram-negative bacterial infections. FEBS J..

[40] Nicholson TF, Watts KM, Hunstad DA (2009). OmpA of uropathogenic *Escherichia coli* promotes postinvasion pathogenesis of cystitis. Infect. Immun..

[41] Wang Y, Kim KS (2002). Role of OmpA and IbeB in *Escherichia coli* K1 invasion of brain microvascular endothelial cells in vitro and in vivo. Pediatr. Res..

[42] Huang SH (1999). Identification and characterization of an *Escherichia coli* invasion gene locus, ibeB, required for penetration of brain microvascular endothelial cells. Infect. Immun..

[43] Ishii S, Ksoll WB, Hicks RE, Sadowsky MJ (2006). Presence and growth of naturalized *Escherichia coli* in temperate soils from lake superior watersheds. Appl. Environ. Microbiol..

[44] Prasadarao NV (1996). Outer membrane protein A of *Escherichia coli* contributes to invasion of brain microvascular endothelial cells. Infect. Immun..

[45] Pakbin B, Brück WM, Rossen JWA (2021). Virulence factors of enteric pathogenic *Escherichia coli*: A review. Int. J. Mol. Sci..

[46] Sarowska J (2019). Virulence factors, prevalence and potential transmission of extraintestinal pathogenic *Escherichia coli* isolated from different sources: Recent reports. Gut Pathog..

[47] Kaper JB, Nataro JP, Mobley HLT (2004). Pathogenic *Escherichia coli*. Nat. Rev. Microbiol..

[48] Kim KJ, Elliott SJ, Di Cello F, Stins MF, Kim KS (2003). The K1 capsule modulates trafficking of *E. coli*-containing vacuoles and enhances intracellular bacterial survival in human brain microvascular endothelial cells. Cell Microbiol..

[49] King JE, Aal Owaif HA, Jia J, Roberts IS (2015). Phenotypic heterogeneity in expression of the K1 polysaccharide capsule of uropathogenic *Escherichia coli* and downregulation of the capsule genes during growth in urine. Infect. Immun..

[50] Zeng Q (2017). Probiotic mixture golden Bifido prevents neonatal *Escherichia coli* K1 translocation via enhancing intestinal defense. Front. Microbiol..

[51] Cordeiro MA, Werle CH, Milanez GP, Yano T (2016). Curli fimbria: An *Escherichia coli* adhesin associated with human cystitis. Braz. J. Microbiol..

[52] Klemm P, Schembri M (2004). Type 1 fimbriae, curli, and antigen 43: Adhesion, colonization, and biofilm formation. EcoSal Plus.

[53] Brannon JR (2020). Invasion of vaginal epithelial cells by uropathogenic *Escherichia coli*. Nat. Commun..

[54] Yao Y (2009). The type III secretion system is involved in the invasion and intracellular survival of *Escherichia coli* K1 in human brain microvascular endothelial cells. FEMS Microbiol. Lett..

[55] Wang S (2016). *Escherichia coli* type III secretion system 2 ATPase EivC Is involved in the motility and virulence of avian pathogenic *Escherichia coli*. Front. Microbiol..

[56] Cowley LA, Oresegun DR, Chattaway MA, Dallman TJ, Jenkins C (2018). Phylogenetic comparison of enteroinvasive *Escherichia coli* isolated from cases of diarrhoeal disease in England, 2005–2016. J. Med. Microbiol..

[57] Dhakal BK, Mulvey MA (2009). Uropathogenic *Escherichia coli* invades host cells via an HDAC6-modulated microtubule-dependent pathway. J. Biol. Chem..

